# Evaluation of sIgE Qualitative Conversion and Clinical Response to HDMs Sublingual Immunotherapy: Insights from Three Immunoassays

**DOI:** 10.3390/biom16060905

**Published:** 2026-06-18

**Authors:** Tarek Gheith, Sherihan M. Rohayem, Atef Taha El Bahrawy, Amira E. Mesbah, Safaa Gaber Aly Salem, Noha M. Hammad

**Affiliations:** 1 Department of Medical Microbiology and Immunology, Faculty of Medicine, Zagazig University, Zagazig 44519, Egypt; tarekgheith84@gmail.com; 2 Department of Medical Microbiology and Immunology, Faculty of Medicine, Portsaid University, Portsaid 42511, Egypt; sherihanrohayem@med.psu.edu.eg; 3 Department of Otorhinolaryngology, Faculty of Medicine, Zagazig University, Zagazig 44519, Egypt; aelbahrawi@gmail.com; 4 Department of Acarology, Plant Protection Research Institute, Agricultural Research Center, Giza 12611, Egypt; amiramesbah80@gmail.com; 5 Department of Maternity, Obst & Gyn Nursing, College of Nursing, Princess Nourah bint Abdulrahman University, Riyadh 11671, Saudi Arabia; sgsalem@pnu.edu.sa; 6 Al Jouf Regional Laboratory, Shared Services, Ministry of Health, Sakaka 73244, Saudi Arabia

**Keywords:** CLIA, ImmunoCAP, immunoblot, qualitative IgE conversion, SLIT

## Abstract

Background: Sublingual immunotherapy (SLIT) is an effective treatment for house dust mite (HDM)-induced allergic rhinitis (AR); however, the significance of qualitative changes in specific IgE (sIgE) remains unclear. This study evaluated post-treatment changes in sIgE reactivity and compared the performance of three immunoassays. Methods: In this prospective study, monosensitized patients with HDM-induced AR were identified using skin prick testing and followed for 12 months after SLIT. Serum sIgE levels were assessed at baseline and after treatment using immunoblot, chemiluminescent immunoassay (CLIA), and ImmunoCAP as the reference method. Qualitative changes in sIgE reactivity were analyzed. Clinical response was assessed using the total nasal symptom score (TNSS), and total IgE (tIgE) levels were measured. Results: At baseline, HDM-sIgE reactivity was detected in 85.7%, 82.1%, and 92.9% of patients by immunoblot, CLIA, and ImmunoCAP, respectively. Following SLIT, a significant qualitative conversion to non-reactive status was observed across all assays (*p* < 0.001). Conversion rates were 94.0% for immunoblot, 83.3% for CLIA, and 100.0% for ImmunoCAP. Significant improvements in TNSS and reductions in tIgE were also observed. Conclusions: SLIT induces a marked qualitative reduction in HDM-sIgE reactivity, with complete serological conversion detected by ImmunoCAP. Although the immunoassays showed comparable rates of HDM-sIgE detection, their agreement in classifying individual patients differed, indicating variability in assay performance. Qualitative assessment of sIgE may provide a clinically meaningful approach for monitoring immunotherapy response.

## 1. Introduction

Allergic rhinitis (AR) is a highly prevalent IgE-mediated inflammatory disorder, affecting an estimated 10–40% of the global population [1]. AR results from type I hypersensitivity reactions, in which allergen exposure triggers T helper 2-driven production of allergen-specific IgE (sIgE), mast cell activation, and the release of mediators, leading to nasal congestion, sneezing, rhinorrhea, and itching [2]. Among the major aeroallergens, house dust mites (HDMs)—particularly *Dermatophagoides pteronyssinus* and *Dermatophagoides farinae*—represent one of the most common triggers worldwide [3]. HDM fecal particles, rich in proteolytic and immunostimulatory proteins, readily penetrate epithelial surfaces and initiate robust atopic inflammation [4].

Sublingual immunotherapy (SLIT) has emerged as a safe and effective disease-modifying treatment for HDM-induced AR, aiming to induce immunological tolerance through repeated administration of standardized allergen extracts [5,6]. However, reliable biomarkers for monitoring response to SLIT remain an area of ongoing investigation [7]. Total IgE (tIgE) levels fluctuate widely and correlate poorly with symptoms, and quantitative sIgE measurements do not consistently reflect clinical improvement [8]. Consequently, clinicians continue to rely primarily on symptom scores rather than immunologic markers when evaluating SLIT effectiveness.

Accurate assessment of HDM sensitization remains essential for diagnosis and for selecting appropriate candidates for immunotherapy. Skin prick testing (SPT) is considered the first-line diagnostic tool because of its rapid results and accessibility [9]; however, it may be contraindicated in selected patients and is subject to operator-dependent variability. In vitro assays, including immunoblot, chemiluminescent immunoassay (CLIA), and ImmunoCAP, offer quantitative and qualitative alternatives for measuring circulating sIgE and are increasingly incorporated into clinical decision-making [10,11,12]. Despite their widespread use, these assays differ in analytical principles, allergen presentation, and sensitivity profiles, which may influence both diagnostic outcomes and interpretation during immunotherapy follow-up.

Given these considerations, there remains a need to clarify how different sIgE assays perform in detecting HDM sensitization and to determine whether qualitative changes in sIgE reactivity correspond to clinical improvement during SLIT. To address this gap, we prospectively evaluated three widely used immunoassays—immunoblot, CLIA, and ImmunoCAP—alongside SPT and the total nasal symptom score (TNSS) in HDM-sensitized AR patients undergoing 12 months of SLIT. Particular emphasis was placed on qualitative IgE conversion as a potentially simple and clinically informative marker of treatment-related immunologic change. Accordingly, the present study aimed to characterize qualitative HDM-specific IgE conversion across these assays, assess their comparative performance before and after SLIT, and examine whether such conversion aligns with improvement in TNSS.

## 2. Materials and Methods

### 2.1. Patients

**Figure 1:**
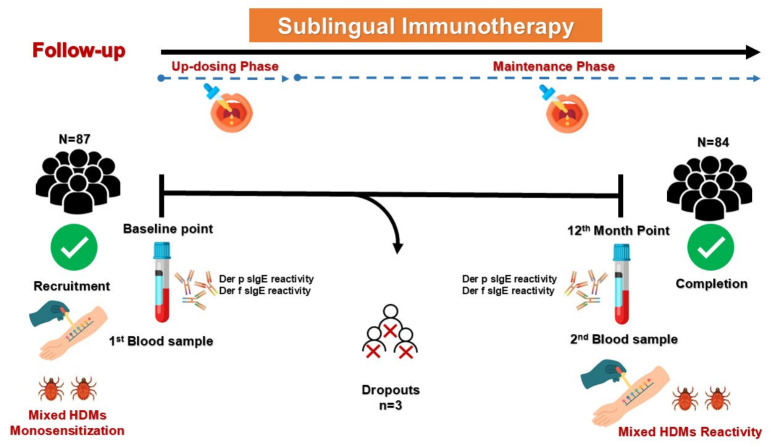
Schematic representation of the study design and participant flow. A total of 87 patients with allergic rhinitis sensitized to house dust mites (HDMs) were screened using skin prick testing (SPT). Three patients were lost to follow-up, leaving 84 participants who completed 12 months of sublingual immunotherapy and were included in the final analysis. Clinical outcomes were assessed at baseline and after 12 months of treatment using the total nasal symptom score. Immunologic evaluation included measurement of HDM-specific immunoglobulin E (sIgE) using immunoblot assay, chemiluminescent immunoassay, and the ImmunoCAP at both time points.

This prospective cohort study was conducted between November 2020 and June 2023. Patients were recruited from individuals attending the Ear, Nose, and Throat outpatient clinic at Zagazig University Hospitals (Zagazig, Egypt) for evaluation and management of AR. All participants underwent a comprehensive clinical evaluation, including assessment of sociodemographic characteristics and medical history. The diagnosis of AR was established in accordance with the Allergic Rhinitis and Its Impact on Asthma (ARIA) guidelines [13]. SPT was subsequently performed at the Allergy and Immunology Unit, Zagazig University Hospitals (Zagazig, Egypt) to identify individuals monosensitized to mixed HDM allergens. Eligible patients were selected using systematic random sampling. The study flow, including patient recruitment and follow-up, is shown in Figure 1.

The sample size for this study was calculated using OpenEpi 3.01. Based on previously published data, the expected diagnostic sensitivity of the CLIA (Immulite) assay was assumed to be 71.4% [14]. A precision of 10% and a confidence level of 95% were applied. Accordingly, OpenEpi estimated that 79 HDM-positive participants (confirmed by SPT) were required to achieve the desired level of precision. To compensate for a potential dropout rate of 10%, the sample size was increased to 87 participants. This sample size also exceeded the requirement for the immunoblot assay (expected sensitivity of 83.0%) [10], ensuring adequate precision for all diagnostic comparisons.

Exclusion criteria for participation in this study included pregnancy and lactation, chronic illnesses, malignancy, and autoimmune diseases; inability to discontinue antihistamines at least one week before SPT; use of oral and/or intranasal corticosteroids or anti-IgE medications; non-IgE-mediated AR; dermographism; severe eczema; prior specific immunotherapy; and patient refusal.

### 2.2. Total Nasal Symptom Score

The total nasal symptom score was calculated at the time of diagnosis and after a 12-month follow-up period. The TNSS is defined as the sum of scores for each of nasal congestion, sneezing, nasal itching, and rhinorrhea at each time point, using a four-point scale (0–3). The total score ranges from 0 to 12 and is calculated by summing the individual symptom scores [15].

### 2.3. Skin Prick Test

Skin prick testing was performed by placing a drop of allergen extract on the volar surface of the forearm. Standardized allergen extracts (Omega Laboratory Ltd., Montreal, QC, Canada) for 12 common aeroallergens (cat epithelium, dog epithelium, mixed feather, mixed pollens, mixed grasses, mixed molds, mixed mites, house dust, latex, horse epithelium, mosquito, and cockroach) were used (Supplementary Figure S1).

Histamine phosphate solution (10 mg/mL histamine base) was used as a positive control. A negative control consisting of glycerinated normal saline (NaCl 0.9% *w*/*v*) was also included [16]. The test was considered positive when the wheal diameter was at least 3 mm greater than that of the negative control [17].

### 2.4. Blood Sampling

Five mL of peripheral blood was collected from each patient by venipuncture into serum tubes containing a clot activator. After centrifugation, the serum was aliquoted and stored at −20 °C for subsequent measurement of serum total and HDM-specific IgE (sIgE) at each time point.

### 2.5. Measurement of tIgE Serum Level

Serum tIgE level was measured using automated COBAS e (Cobas e 411 analyzer test number 630, Roch, Tokyo, Japan). The normal threshold of serum tIgE for adults was 100 IU/mL.

### 2.6. In Vitro Measurement of Serum-Specific IgE

HDM-sIgE was assessed using different immunoassay platforms. Quantitative sIgE values were not included in the analysis, as the assays use distinct measurement scales and calibration systems, limiting direct comparability. Therefore, results were standardized to qualitative outcomes (reactive/non-reactive).

#### 2.6.1. Immunoblot Assay

Serum sIgE for 30 aeroallergens, including HDMs (Desert palm pollen, elder, mesquite tree, birch, Bermuda grass, timothy grass, rye pollen, mixed grasses, ragweed/mugwort mix, plantain, *Chenopodium album*, Russian thistle, sunflower, *Cladosporium herbarum*, *Alternaria alternata*, *Penicillium notatum*, *Aspergillus niger*, *Aspergillus fumigatus*, *Candida albicans*, cat epithelium, dog epithelium, feather mix, house dust, *Dermatophagoides farinae*, *Dermatophagoides pteronyssinus*, honey bee venom, cockroach, common wasp venom, mosquito, and latex) was measured using an immunoblot assay (Alleisa Screen^®^ system, MEDIWISS Analytic GmbH, Moers, Germany). Serum sIgE was analyzed using a rapid scanner [16]. An sIgE level ≥ 0.35 IU/mL was considered to be positive according to the manufacturer’s protocol.

#### 2.6.2. Chemiluminescent Immunoassay

Serum sIgE for HDMs: *D. pteronyssinus* and *D. farina* (Catalog No. D1L40 and D2 Catalog No. D2L4, respectively) was measured using the 3g Allergy™ sIgE CLIA on the Immulite 2000/XPi system (Siemens, Munich, Germany). An sIgE serum level ≥ 0.35 kU/L was considered positive according to the manufacturer’s recommendation.

#### 2.6.3. ImmunoCAP Test

All serum samples were tested by serum-specific ImmunoCAP IgE for *D. pteronyssinus* (ImmunoCAP Allergen d1, House dust mite) and *D. farina* (ImmunoCAP Allergen d2, House dust mite) on the ImmunoCAP™ Phadia 250 instrument (Thermo Fisher Scientific™, Waltham, MA, USA). A serum sIgE level ≥ 0.35 kUA/L was considered positive according to the manufacturer’s recommendation.

### 2.7. Immunotherapy

After diagnosis, all participants started SLIT. SLIT was prepared from a standardized allergenic mite extract mixture (*D. pteronyssinus*; 50% and *D. farina*; 50% [10,000 AU/mL]) (Omega Laboratory Ltd., Montreal, QC, Canada). The allergenic mite extract was aseptically diluted in sterile 10% glycerol–saline solution containing 0.5% phenol under aseptic conditions. The up-dosing and maintenance phases of SLIT were scheduled as demonstrated in Supplementary Tables S1 and S2.

### 2.8. Follow-Up

After a 12-month period from the initiation of SLIT, all patients were re-evaluated clinically by TNSS, SPT, measurement of serum tIgE and HDM-sIgE by immunoblot assay, CLIA, and ImmunoCAP.

### 2.9. Statistical Analysis

Patients who completed the study period were included in the statistical analysis. Data were entered and analyzed using the Statistical Package for the Social Sciences (SPSS), version 25 (IBM Corp., Armonk, NY, USA). Qualitative data are presented as frequencies and percentages, while quantitative data are expressed as mean ± standard deviation (SD) and median (range).

Differences between quantitative variables within the same group were assessed using the paired *t*-test or the Wilcoxon signed-rank test for parametric and non-parametric data, respectively. The McNemar test was used to assess differences between paired qualitative variables. Receiver operating characteristic (ROC) curve analysis was performed to evaluate diagnostic performance in comparison with the reference method. A *p*-value ≤ 0.05 was considered statistically significant.

## 3. Results

### 3.1. Study Population

A total of 87 patients with AR who tested positive for mixed HDM extract by SPT were initially recruited. Of these, 84 patients completed the 12-month follow-up period after SLIT and were included in the final analysis. The mean age of the study population was 28.9 ± 6.6 years. Males accounted for 55.0% of the participants, while females represented 45.0%.

### 3.2. Diagnostic Performance for HDM Sensitization

**Figure 2:**
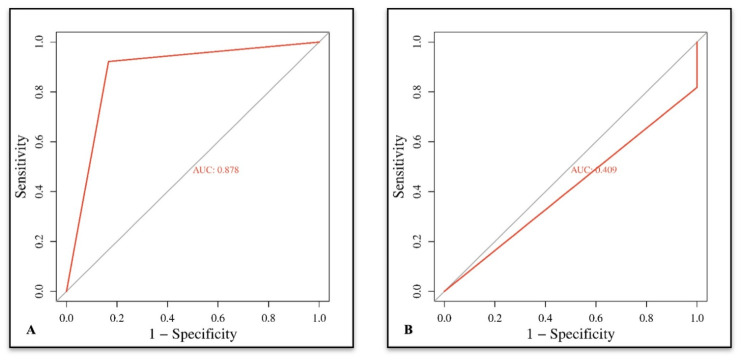
Receiver operating characteristic (ROC) curve analysis of baseline HDM-specific IgE reactivity using (**A**) immunoblot assay and (**B**) chemiluminescent immunoassay (CLIA), with ImmunoCAP used as the reference standard.

The diagnostic performance of the evaluated assays was assessed for the detection of HDM sensitization—defined as positivity to at least one of *Dermatophagoides pteronyssinus* or *Dermatophagoides farinae*—using ImmunoCAP as the reference standard (Figure 2).

The immunoblot assay demonstrated a sensitivity of 91.0% and a specificity of 83.3%, while CLIA showed a sensitivity of 83.3% and a specificity of 33.3%. Agreement analysis at baseline demonstrated moderate agreement for immunoblot (κ = 0.509) and slight agreement for CLIA (κ = −0.114) compared with ImmunoCAP. Kappa statistics were not calculated post-SLIT due to the lack of variability in ImmunoCAP results, as all patients became non-reactive. Although differences in positivity rates were observed among the evaluated assays, these differences did not reach statistical significance (Cochran’s Q test, *p* = 0.089) (Table 1).

Although comparison between SPT and ImmunoCAP was not a predefined study objective, baseline data enabled the proportion of SPT-positive patients confirmed as HDM-sensitized by ImmunoCAP. At baseline, all 84 enrolled patients were SPT-positive according to the inclusion criteria. Among these, 78 patients (92.9%) were confirmed to be HDM-sensitized by ImmunoCAP, while 6 patients (7.1%) tested negative.

Accordingly, the positive predictive value of SPT for HDM sensitization was 92.9%, and the overall concordance rate between SPT and ImmunoCAP was also 92.9%. Sensitivity, specificity, negative predictive value, and kappa statistics could not be calculated due to the absence of SPT-negative participants.

### 3.3. Qualitative Changes in sIgE Following SLIT

**Figure 3:**
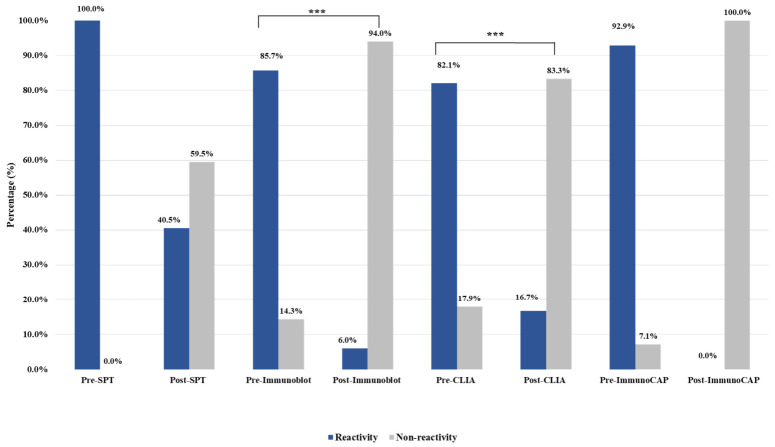
Qualitative conversion of HDM-sIgE reactivity. The figure illustrates the percentage of patients classified as reactive and non-reactive before (pre) and after 12 months of sublingual immunotherapy (post), as assessed by skin prick test (SPT), immunoblot, chemiluminescent immunoassay (CLIA), and ImmunoCAP. A marked increase in the proportion of non-reactive cases was observed across all methods, with the highest conversion rate detected by ImmunoCAP (100%). Statistical significance is indicated by *** (*p* < 0.001).

Among patients identified as HDM-sensitized by SPT, baseline HDM-sIgE reactivity was detected in 85.7% and 82.1% of patients using the immunoblot assay and CLIA, respectively, while 92.9% tested positive using ImmunoCAP (Figure 3).

A marked reduction in HDM-sIgE reactivity was observed following SLIT across all evaluated assays (Figure 3). The proportion of patients demonstrating non-reactive results increased significantly from baseline in both the immunoblot (94.0%) and CLIA (83.3%) assays (*p* < 0.001 for both). Notably, complete serological conversion to non-reactivity (100.0%) was observed using ImmunoCAP after 12 months of treatment (Table 2).

### 3.4. Clinical Response to SLIT (TNSS)

**Figure 4:**
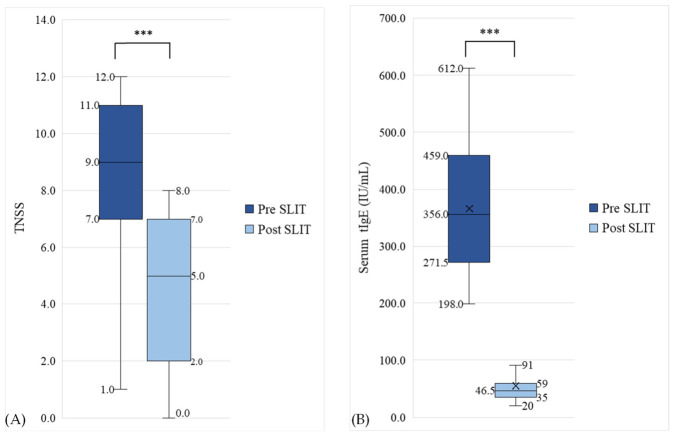
Boxplots showing significant reductions in (**A**) TNSS and (**B**) serum total IgE (tIgE, IU/mL) before (Pre SLIT) and after 12 months of sublingual immunotherapy (Post SLIT). Data were analyzed using paired *t*-test and the Wilcoxon signed-rank test for TNSS and tIgE, respectively. Statistical significance is indicated by *** (*p* < 0.001). The symbol × denotes the mean value.

A significant improvement in clinical symptoms was observed following SLIT, as reflected by a reduction in the TNSS. The mean TNSS decreased from 9.1 ± 2.0 at baseline to 5.0 ± 2.1 after 12 months of treatment (*p* < 0.001), resulting in a significant clinical response to therapy, as illustrated in Figure 4A.

### 3.5. Changes in Serum tIgE Levels

Serum tIgE levels demonstrated a statistically significant reduction following SLIT. The median tIgE level decreased from 356.0 IU/mL (range: 198.0–612.0) at baseline to 46.5 IU/mL (range: 20.0–360.0) after 12 months of treatment (*p* < 0.001).

At baseline, serum tIgE levels differed significantly between HDM-sIgE-reactive and non-reactive patients when reactivity was determined by the immunoblot assay and the CLIA (*p* = 0.048 and *p* = 0.01, respectively). In contrast, no significant difference in tIgE levels was observed between reactive and non-reactive groups when classification was based on ImmunoCAP results (*p* = 0.90) (Supplementary Table S3).

After 12 months of SLIT, serum tIgE levels did not differ significantly between HDM-sIgE reactive and non-reactive patients when assessed by SPT, immunoblot assay, or CLIA (*p* = 0.30, *p* = 0.80, and *p* = 1.00, respectively) (Supplementary Table S4).

### 3.6. Correlation Between TNSS and tIgE Levels

**Figure 5:**
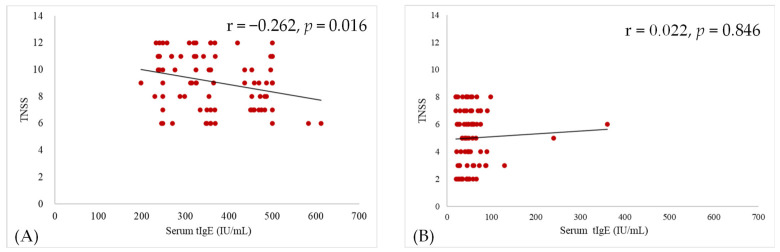
Scatter plots show the relationship between Total Nasal Symptom Score (TNSS) and serum total IgE (tIgE) levels at (**A**) baseline and (**B**) after 12 months of sublingual immunotherapy (SLIT).

At baseline, TNSS was weakly and negatively correlated with serum tIgE levels (r = −0.262, *p* = 0.016). However, after SLIT, no significant correlation was detected (r = 0.022, *p* = 0.846) (Figure 5).

### 3.7. Association Between TNSS Improvement and Qualitative sIgE Conversion

To investigate the relationship between clinical response and qualitative immunological changes, improvement in TNSS (ΔTNSS) was compared between patients who achieved qualitative sIgE conversion and those who remained reactive after 12 months of SLIT.

For CLIA, patients who converted to a non-reactive status demonstrated a significantly greater reduction in TNSS, with a median ΔTNSS of 4.0 (−2.0–10.0), compared with 2.0 (0.0–8.0) in non-converters (*p* = 0.023). This finding indicates a significant association between loss of HDM-sIgE reactivity and clinical improvement.

In contrast, patients classified by immunoblot showed a median ΔTNSS of 4.0 (−2.0–10.0) among converters and 3.0 (1.0–7.0) among non-converters. Although a greater reduction in TNSS was observed among immunoblot converters compared with non-converters, this difference did not reach statistical significance (*p* = 0.544), likely due to the small number of non-converters identified by immunoblot (*n* = 5) (Table 3).

As all patients became non-reactive when assessed by ImmunoCAP, comparative analysis was not feasible for this method.

## 4. Discussion

Allergic rhinitis is a common IgE-mediated inflammatory disorder characterized by nasal and ocular symptoms, with HDM representing a major perennial allergen worldwide [18,19]. Accurate diagnosis and monitoring of AR remain challenging, particularly during immunotherapy, as quantitative IgE levels often show inconsistent correlation with symptoms [20,21]. In this context, the presence of sIgE reflects sensitization rather than confirmed allergic disease, and its clinical relevance depends on correlation with patient symptoms. Therefore, reliance on absolute sIgE concentrations alone may be insufficient for assessing treatment response. Accordingly, the present prospective study evaluated qualitative changes in HDM-sIgE reactivity using ImmunoCAP, immunoblot, and CLIA over 12 months of SLIT in HDM-monosensitized AR patients, and explored their association with clinical outcomes.

In this study, SPT demonstrated high concordance with ImmunoCAP, with 92.9% of SPT-positive patients confirmed as sensitized by ImmunoCAP. However, as only SPT-positive individuals were included, the analysis was limited to the calculation of the positive predictive value and observed agreement, while the sensitivity, specificity, negative predictive value, and kappa statistics could not be reliably assessed. Therefore, these findings should be interpreted as descriptive rather than definitive measures of diagnostic performance.

When compared with ImmunoCAP, the immunoblot assay demonstrated high sensitivity (91.0%), specificity (83.3%), and overall diagnostic accuracy (90.5%), consistent with previous studies reporting moderate agreement between immunoblot platforms and ImmunoCAP [22,23]. In contrast, CLIA exhibited limited specificity (33.3%), highlighting variability between assay platforms. Collectively, these findings support the use of immunoblot as a reliable alternative in settings where ImmunoCAP is unavailable, while emphasizing the need to account for inter-assay differences during interpretation.

Although baseline positivity rates were similar among the three immunoassays, agreement varied. Immunoblot showed moderate agreement with ImmunoCAP, consistent with previous reports of acceptable correlation [10,22], whereas CLIA exhibited only slight agreement, in line with prior observations [24]. These findings indicate that similar positivity rates at the population level do not necessarily translate into concordant classification at the individual level, underscoring the importance of considering assay-specific performance when interpreting sIgE results, particularly in the context of treatment monitoring.

While ImmunoCAP remains the reference method [25], immunoblot may represent a practical and cost-effective alternative for routine use [26]. In contrast, results obtained by CLIA should be interpreted with caution due to its lower specificity. It is well recognized that CLIA, such as IMMULITE, may overestimate sIgE levels due to their analytical characteristics, whereas ImmunoCAP aligns more closely with true IgE binding based on standardized monoclonal systems [27,28]. This analytical discrepancy likely contributed to the lower specificity and weaker agreement observed between the CLIA and ImmunoCAP in our diagnostic evaluation. In addition, descriptive comparison between SPT and ImmunoCAP—although not a predefined study objective—showed high baseline concordance, consistent with the SPT-positive inclusion criterion.

A significant improvement in clinical symptoms was observed after 12 months of SLIT, as reflected by a marked reduction in TNSS. This finding is consistent with previous studies demonstrating the effectiveness of SLIT in reducing symptom burden and improving clinical outcomes in patients with HDM-induced AR [29,30,31,32,33].

Baseline serum tIgE levels were elevated in our cohort and decreased following treatment. However, reductions in tIgE during immunotherapy have been inconsistently reported in the literature [34,35,36], with several studies demonstrating minimal or no change [31,32,33]. This variability may be influenced by factors such as treatment duration and timing of sample collection [20].

Despite the observed reduction, the clinical relevance of tIgE remained limited. The weak inverse correlation between TNSS and tIgE at baseline suggests that tIgE contributed minimally to symptom severity. Following SLIT, this relationship was no longer observed, reinforcing the limited utility of tIgE as a biomarker for monitoring treatment response during immunotherapy [36,37]. Accordingly, this finding should be interpreted with caution, as tIgE reflects overall atopic status rather than a specific marker of treatment efficacy. While tIgE may retain value as a general screening indicator, it lacks sensitivity for monitoring response to SLIT [31].

At baseline, patients classified as HDM-sIgE reactive by immunoblot and CLIA exhibited higher serum tIgE levels compared with non-reactive individuals, consistent with the established association between elevated tIgE and atopic sensitization to HDMs [37,38], suggesting that the results of these assays may be influenced by overall atopic burden rather than solely reflecting allergen-specific sensitization. In contrast, no corresponding difference in serum tIgE levels was observed when patients were classified using ImmunoCAP. This discrepancy suggests that ImmunoCAP may be less influenced by variations in overall IgE levels and may more specifically reflect allergen-directed sensitization. Such differences likely relate to variations in assay design and analytical characteristics, including the semi-quantitative nature of immunoblot and the differing sensitivity profiles of CLIAs. Taken together, these findings indicate that the relationship between tIgE and allergen-sIgE is not uniform across diagnostic platforms and may be influenced by both methodological and biological factors. Consequently, the observed associations should be carefully interpreted, as they may partly reflect assay-related characteristics rather than solely underlying immunological differences.

Previous studies have reported variable effects of SLIT on serum sIgE, ranging from no significant change—even after more than three years of treatment—[32] into significant reductions [33,35,36] without parallel changes in IgG1 or IgG4 antibodies [39,40,41]. In contrast, the present study demonstrated a clear qualitative decline in HDM-sIgE reactivity across all assays, accompanied by clinically meaningful improvement.

To better interpret these findings, it is important to distinguish between sensitization and clinical allergy. Sensitization reflects immunologic recognition, defined by the presence of detectable allergen sIgE or a positive skin test, even in the absence of symptoms, whereas clinical allergy refers to symptom-inducing reactivity upon allergen exposure [42,43]. Accordingly, sIgE results should always be considered in the context of clinical history and patient presentation. Moreover, commonly used thresholds such as 0.35 kU/L primarily reflect analytical detection limits rather than definitive indicators of clinical reactivity. Advances in assay sensitivity have demonstrated that lower sIgE levels may also carry clinical relevance in certain individuals, emphasizing the complexity of interpreting IgE measurements [43].

One of the most striking observations in this study was the complete absence of HDM-sIgE detected by ImmunoCAP after 12 months of SLIT. Although reductions in sIgE are expected during immunotherapy, complete negativization within the first year is not consistently reported. This finding likely reflects, at least in part, the qualitative interpretation of results based on assay detection thresholds, whereby reductions below the cutoff are recorded as negative rather than representing complete immunological elimination. In addition, the homogeneity of our monosensitized cohort and the use of a standardized HDM extract may have contributed to the observed response. Moreover, the high level of qualitative conversion observed, particularly with ImmunoCAP, suggests measurable immunologic modulation in this specific cohort. While these observations align with known mechanisms of immunotherapy—such as regulatory T-cell induction and IgG4 production—[44], the present study was not designed to confirm these pathways, and the immunological interpretations should be considered supportive rather than definitive.

While qualitative classification has inherent limitations compared with quantitative analysis [43], it provides a practical approach for standardizing comparisons across immunoassays with differing measurement scales, calibration systems, and degrees of quantification. Therefore, the observed findings likely represent a substantial reduction in sIgE levels rather than a complete absence and should be interpreted within this methodological context.

Despite the observed ImmunoCAP seronegativity, SPT remained positive in 40.5% of patients. This discrepancy likely reflects mast-cell-bound IgE in the skin, which declines more slowly due to delayed effector cell turnover [9,44]. Consistent with previous literature, SPT reactivity did not correlate with clinical improvement, supporting its limited utility as a biomarker for monitoring immunotherapy response [9,45].

The significant association between TNSS improvement and qualitative IgE conversion was observed when assessed using the CLIA, suggesting that loss of measurable sIgE reactivity corresponds with meaningful clinical benefit. The higher number of residual reactive cases identified by this assay likely reflects its known tendency to overestimate sIgE [27,28], resulting in a slower apparent conversion rate and preserving sufficient variability to detect a statistical association with symptom improvement. In contrast, the near-complete conversion observed with immunoblot and the complete seronegativity detected by ImmunoCAP produced a ceiling effect, leaving too few non-converters for meaningful subgroup comparison. Consequently, the absence of statistical significance for immunoblot reflects limited variability rather than a true lack of association with clinical response.

ImmunoCAP is recommended as the primary assay for diagnosing HDM sensitization because of its superior analytical performance [46], and the complete conversion observed in this study underscores its high sensitivity to treatment-related immunologic changes. Meanwhile, the CLIA—by retaining a larger subgroup of non-converters—may offer additional value in identifying partial or slower responders, whereas immunoblot, despite strong diagnostic performance, may be less informative for SLIT follow-up due to near-complete conversion. Together, these findings indicate that qualitative sIgE conversion, particularly when assessed using assays that maintain adequate variability, may serve as a clinically meaningful indicator of SLIT response.

Overall, these findings support the clinical utility of SLIT in monosensitized HDM-induced AR [39,47] and confirm the reliability of immunoblotting as a diagnostic tool when ImmunoCAP is unavailable or cost-prohibitive. Moreover, the use of qualitative sIgE conversion offers a practical and clinically interpretable marker of immunologic change during SLIT. However, validation in larger and more diverse populations is warranted before routine clinical implementation. Future studies should incorporate larger, multicenter cohorts with extended follow-up and explore biomarkers such as IgG4, basophil activation, and cytokine patterns to identify predictors of rapid response.

## 5. Strengths and Limitations

This study has several strengths. Its prospective design and paired pre- and post-treatment assessments enabled direct evaluation of both clinical outcomes and immunological changes following 12 months of SLIT. The use of three commonly available diagnostic immunoassays—immunoblot, CLIA, and ImmunoCAP—allowed for a comprehensive comparison of in vitro platforms used in routine practice. Focusing on a monosensitized HDM cohort minimized heterogeneity and facilitated clearer interpretation of treatment-related effects. Although the study was conducted at a single center, the Allergy and Immunology Unit at Zagazig University Hospitals serves patients from at least five governorates and is one of the few governmental referral centers offering specialized allergy testing in Egypt, thereby enhancing the regional representativeness of the study population. Integrating qualitative sIgE outcomes with clinical symptom scores further strengthened the evaluation of SLIT response.

Nevertheless, several limitations should be considered. Inclusion was restricted to SPT-positive patients, precluding full diagnostic accuracy evaluation of SPT and potentially limiting generalizability to individuals with discordant skin or serologic findings. The absence of a control group limits causal inference regarding the magnitude of SLIT-related changes. The 12-month follow-up period may not fully capture the long-term durability of qualitative IgE conversion or sustained clinical improvement. Qualitative IgE assessment, while clinically intuitive, does not quantify the magnitude of immunologic change. In addition, although the immunoassays used are capable of generating quantitative sIgE values, direct comparison of titers was not performed due to differences in measurement scales and calibration systems across platforms, which may further limit detailed assessment of immunological changes. Differences in assay methodology likely contributed to variability in conversion rates, and the near-complete loss of sIgE reactivity observed with ImmunoCAP and immunoblot introduced a ceiling effect, reducing variability and limiting the ability to examine associations between conversion and TNSS improvement. Finally, as a single-center study, the findings may not be fully generalizable to populations with different sensitization patterns or environmental exposures despite the broad regional catchment of the center.

## 6. Conclusions

In this prospective cohort, 12 months of SLIT resulted in meaningful clinical improvement and substantial qualitative reductions in HDM-sIgE across all evaluated immunoassays. Although conversion patterns differed between platforms, ImmunoCAP showed complete qualitative loss of sIgE, immunoblot demonstrated near-complete conversion, and the CLIA retained greater variability, allowing clearer discrimination of partial responders. These assay-dependent differences highlight the importance of considering test characteristics and underlying sensitization profiles when interpreting immunologic outcomes. Given that clinical populations often show heterogeneous sensitization patterns, including mono and polysensitization, the behavior of qualitative IgE conversion observed in this homogeneous cohort may differ in more diverse settings. Therefore, while qualitative sIgE conversion appears to be a practical indicator of immunologic change during SLIT, further validation in larger, multicenter cohorts with varied sensitization patterns is required to define its broader applicability and predictive value.

## Figures and Tables

**Table 1:** Diagnostic performance of HDM-sIgE immunoassays compared with ImmunoCAP (reference standard).

HDM-sIgE Immunoassays	AUC	Sensitivity	Specificity	PPV	NPV	Accuracy	Kappa Value	*p*-Value
Immunoblot	0.878	91.0%	83.3%	98.6%	41.7%	90.5%	0.51	<0.001 *
CLIA	0.409	83.3%	33.3%	94.2%	13.3%	79.8%	0.10	0.6

* Significant difference. AUC, area under the curve; CLIA, chemiluminescent immunoassay; HDM, house dust mite; PPV, positive predictive value; NPV, negative predictive value.

**Table 2:** Qualitative changes in HDM-sIgE reactivity before and after SLIT across different diagnostic methods.

Variable	Before SLIT N = 84	After SLIT N = 84	Test of Significance	*p*-Value
Immunoblot Reactive Non-reactive	72 (85.7) 12 (14.3)	5 (6.0) 79 (94.0)	McNemar test	<0.001 *
Chemiluminescence Reactive Non-reactive	69 (82.1) 15 (17.9)	14 (16.7) 70 (83.3)		<0.001 *
ImmunoCAP Reactive Non-reactive	78 (92.9) 6 (7.1)	0 (0.0) 84 (100.0)	-	

* Significant difference.

**Table 3:** Association between clinical improvement (ΔTNSS) and qualitative sIgE conversion.

Variable	∆TNSS Median (Min–Max)	Test of Significance	*p*-Value
SPT Reactive *n* = 34 Non-reactive *n* = 50	4.0 (−1.0–9.0) 4.0 (−2.0–10.0)	Mann–Whitney U test	0.444
CLIA Reactive *n* = 14 Non-reactive *n* = 70	2.0 (0.0–8.0) 4.0 (−2.0–10.0)		0.023 *
Immunoblot Reactive *n* = 5 Non-reactive *n* = 79	3.0 (1.0–7.0) 4.0 (−2.0–10.0)		0.544

* Significant difference.

## Data Availability

The raw data supporting the conclusions of this article will be made available by the authors on request.

## Supplementary Materials

The following supporting information can be downloaded at: https://www.mdpi.com/article/10.3390/biom16060905/s1, Supplementary Figure S1: Skin prick test. (A) All patients were screened for mixed HDMs monosensitization by SPT. 13 common allergen extracts (cat epithelium, dog epithelium, mixed feather, mixed pollens, mixed grass, mixed models, mixed mites, house dust, latex, horse epithelium, bee venom, mosquito and cockroach), normal saline (NaCl 0.9% as negative control, and 10 mg/mL histamine solution as positive control were applied (B) After 20 min, a wheal reaction at least 3 mm in diameter (yellow arrow) more than negative control (white arrow) was considered positive. Red arrow demonstrates positive control reaction. Supplementary Table S1: Sublingual allergen extract treatment schedule (up-dosing phase). Supplementary Table S2: Sublingual allergen extracts treatment schedule (maintenance phase). Supplementary Table S3: Relation between pre-immunotherapy tIgE serum level and HDMs IgE reactivity. Supplementary Table S4: Relation between post-treatment HDMs IgE reactivity and tIgE serum level.
