# Supplementary material for: Evaluation of sIgE Qualitative Conversion and Clinical Response to HDMs Sublingual Immunotherapy: Insights from Three Immunoassays
Source: Biomolecules. 2026 Jun 18;16(6):905. doi: 10.3390/biom16060905 (PMC13296384; doi:10.3390/biom16060905)
Supplement: Supplementary file 1 [file biomolecules-16-00905-s001.zip › biomolecules-4378973-supplementary.pdf]

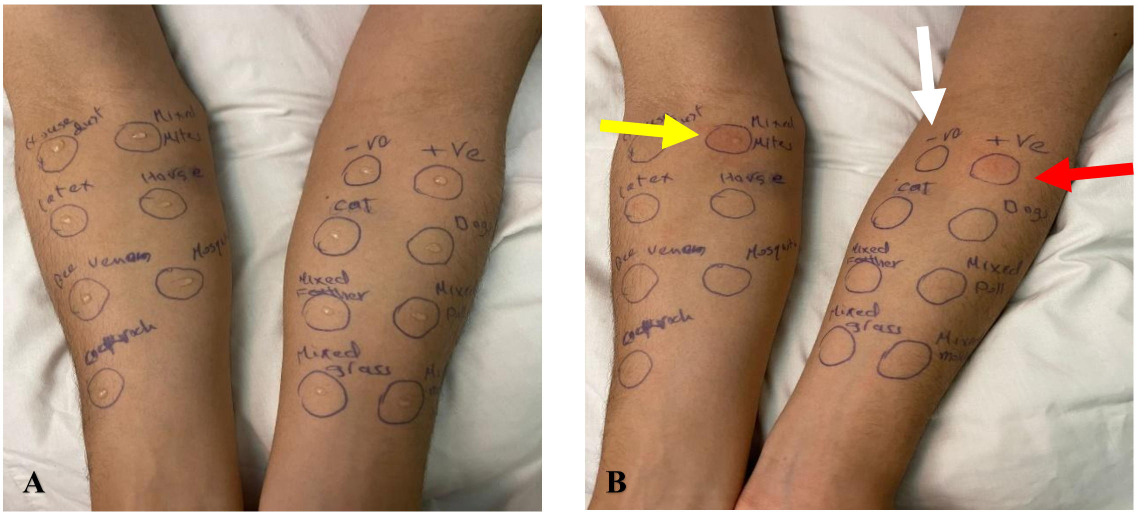

Supplementary Figure S1: Skin prick test. (A) *All patients were screened for mixed HDMs monosensitization by SPT. 13 common allergen extracts (cat epithelium, dog epithelium, mixed feather, mixed pollens, mixed grass, mixed models, mixed mites, house dust, latex, horse epithelium, bee venom, mosquito and cockroach), normal saline (NaCl 0.9% as negative control, and 10 mg/mL histamine solution as positive control were applied* ***(B)*** *After 20 minutes, a wheal reaction at least 3 mm in diameter* ***(yellow arrow)*** *more than negative control* ***(white arrow)*** *was considered positive.* ***Red arrow*** *demonstrates positive control reaction.*

Supplementary Table S1: Sublingual allergen extract treatment schedule (up-dosing phase)

Day	Strength 1R[a]	Strength 2R	Strength 3R	Strength 4R
**First**	2 drops [b]/day (0.18 ml/day)	2 drops/day (0.18 ml/day)	2 drops/day (0.18 ml/day)	4 drops/day (0.24 ml/day)
**Second**	4 drops/day (0.24 ml/day)	4 drops/day (0.24 ml/day)	4 drops/day (0.24 ml/day)	4 drops/day (0.24 ml/day)
**Third**	6 drops/day (0.30 ml/day)	6 drops/day (0.30 ml/day)	6 drops/day (0.30 ml/day)	4 drops/day (0.24 ml/day)
**Fourth**	8 drops/day (0.36 ml/day)	8 drops/day (0.36 ml/day)	8 drops/day (0.36 ml/day)	4 drops/day (0.24 ml/day)
**Fifth**	8 drops/day (0.36 ml/day)	8 drops/day (0.36 ml/day)	8 drops/day (0.36 ml/day)	4 drops/day (0.24 ml/day)
**From sixth day and onwards**	8 drops/day Till the end of the bottle	8 drops/day Till the end of the bottle	8 drops/day Till the end of the bottle	4 drops/day Till the end of the bottle

[a] R refers to the strength of allergen extract solution and is equivalent to 500 AU/mL.
AU; Allergy Unit
[b] the drops should be kept for 2 minutes under the tongue before swallowing

Supplementary Table S2: Sublingual allergen extracts treatment schedule (maintenance phase):

Day	Strength 4R'[a]	Strength 4R''[a]
**First**	4 drops [b] /day<br>(0.24 ml/day)	8 drops /day<br>(0.36 ml/day)
**Second**	6 drops/day<br>(0.30 ml/day)	8 drops/day<br>(0.36 ml/day)
**Third**	8 drops/day<br>(0.36 ml/day)	8 drops/day<br>(0.36 ml/day)
**From the Fourth day onwards**	8 drops/day<br>(0.36 ml/day)<br>Till the end of the bottle	8 drops/day<br>(0.36 ml/day)<br>Till the end of the bottle

[a] *R' refers to the strength of the allergen extract solution and is equivalent to 500 AU/mL. AU; Allergy Unit*

[b] *the drops should be kept for 2 minutes under the tongue before swallowing*

Supplementary Table S3: Relation between pre-immunotherapy total IgE serum level and HDMs IgE reactivity

Variable	Total IgE (IU/mL) Mean ± SD	Test of significance	P-value
**SPT**		-	-
Reactive	366.0 ± 99.3		
**Immunoblot**		Independent samples t-test	**0.048***
Reactive n=72	347.7 ± 95.6		
Non-reactive n=12	313.8 ± 109.7		
**Chemiluminescence**			**0.01***
Reactive n=69	379.0 ± 97.9		
Non-reactive n=15	306.5 ± 85.7		
**ImmunoCAP**			0.9
Reactive n=78	366.3 ± 97.2		
Non-reactive n=6	361.8 ± 135.3		

**Significant difference*

Supplementary Table S4: Relation between post-treatment HDMs IgE reactivity and total IgE serum level

Variable	Total IgE (IU/mL) Mean ± SD	Test of significance	P-value
**SPT**<br>Reactive n=34<br>Non-reactive n=50	51.2 ± 37.2<br>57.6 ± 49.0	Independent samples t-test	0.3
**Immunoblot**<br>Reactive n=5<br>Non-reactive n=79	45.8 ± 8.3<br>55.6 ± 45.8		0.8
**Chemiluminescence**<br>Reactive n=14<br>Non-reactive n=70	59.8 ± 54.0<br>54.1 ± 42.7		1.0
**ImmunoCAP**<br>Reactive n=0<br>Non-reactive n=84	-<br>55.0 ± 44.5	-	-
